# Understanding the Impact of the Gut Microbiome on Mental Health: A Systematic Review

**DOI:** 10.7759/cureus.78100

**Published:** 2025-01-27

**Authors:** Raja Gulfam Shaikh, Animesh Dey, Vindhya Prakash Singh, Anita Khandagle, Baskaran M, Sunil Naik, Asif Hasan

**Affiliations:** 1 Neurology, Mahatma Gandhi Memorial Medical College, Indore, IND; 2 Allied Health Sciences, Brainware University, Kolkata, IND; 3 Psychiatry, Government Medical College, Azamgarh, IND; 4 Nursing, Dr. D. Y. Patil College of Nursing, Pune, IND; 5 Mental Health Nursing, PSG College of Nursing, Coimbatore, IND; 6 Physiology, All India Institute of Medical Sciences, Mangalagiri, Guntur, IND; 7 Psychology, Aligarh Muslim University, Aligarh, IND

**Keywords:** anxiety, bipolar disorder, depression, gut microbiota, mental health, microbiome modulation, schizophrenia

## Abstract

Mental health is a serious issue, with mental health disorders affecting millions of people globally. Gut microbiota has received considerable attention because of its potential role in the pathogenesis of mental health disorders. This systematic review synthesized 15 studies exploring the effects of the gut microbiome on depression, anxiety, schizophrenia, and bipolar disorder, with qualitative and quantitative insights. The studies were conducted in different countries and employed various methods including 16S rRNA sequencing and metagenomic analysis with sample sizes varying from 50 to 600. Some of the key findings were that depression was associated with reduced microbial diversity and high levels of Firmicutes, and anxiety was associated with low levels of short-chain fatty acid (SCFA)-producing bacteria and high levels of Proteobacteria. Schizophrenia was related to endotoxemia and a reduction in the Lactobacillus count whereas bipolar disorder displayed a shift in the Firmicutes/Bacteroidetes ratio. Of interest, probiotics and dietary changes were as effective as drug treatment leading to symptom alleviation in many patients. It was found that depression was linked to less diverse gut bacteria while anxiety was associated with an increase in inflammatory bacteria. People with bipolar disorder were also found to have different gut bacteria patterns. This review also emphasizes the importance of the gut microbiota in the pathophysiology of mental disorders and the promising value of targeting microbiomes in pharmacological treatment approaches.

## Introduction and background

Mental health disorders are on the rise globally and are affecting millions of people of different ages and genders [1]. According to current epidemiological data, approximately one in eight people may experience a mental health problem at some point in their lives [1]. The most predominant mental health situations are anxiety and depression, which account for a significant portion of the global burden of non-communicable diseases [1]. These diseases and disorders have a disabling effect on people's quality of life and create large economic costs for healthcare organizations and society because of the lost production. Thus, the problem of attaining the best psychosocial treatment for many patients remains an urgent one despite the overall progress made in this sphere. Cross-sectional studies based on the Global Burden of Disease Study 2019 show that depressive disorders are among the leading disability-adjusted life year (DALY) causes and require intensive efforts regarding interindividual intervention development. One such promising area of research that has gained considerable attention of late is the gut-brain axis, a two-way communication system between the central nervous system and the gastrointestinal tract via neuronal, hormonal, immunologic, and metabolic signaling [2]. Investigating this gut-brain axis is the hopeful strategy. Trillions of microorganisms known as the gut microbiota, which includes bacteria, viruses, fungi, and archaea, are essential to this system [3]. According to recent studies, the gut microbiota influences several physiological and psychological processes, such as stress, mood regulation, and cognitive performance. For example, gut dysbiosis is a state of dysbiosis in which the beneficial microbes in the gut are fewer in number compared to the pathogenic ones. This imbalance causes dysregulation in critical bodily functions, including immune activation and neurotransmitter production, to the extent of neuroinflammation and the resulting onset of mental health disorders, such as depression and anxiety. Addressing dysbiosis is a promising avenue for developing new therapies [4].

The different ways the gut microbiome affects mental health are numerous and intricate. Studies have discovered that the gut microbiota contributes to the synthesis and breakdown of neurotransmitters, which include dopamine, serotonin, and GABA, that are all involved in mood stability and control. Additionally, it affects the hypothalamic-pituitary-adrenal (HPA) axis, which manages stress response and plays a role in the development of anxiety and mood disorders [5]. Also, another way it is linked is through controlling the inflammation system as inflammatory markers are shown to be associated with mental illnesses including depression. Modulation of these signaling pathways by the gut microbiome is key in linking the gastrointestinal tract and the brain [6]. The research on microbiota-modifying strategies has opened new doors concerning mental disorders. Probiotics, prebiotics, diet changes, and fecal microbiota transplantation (FMT) have been identified as new therapeutic approaches [4]. Probiotics, which can also be referred to as psychobiotics, are defined as live bioactive agents that upon ingestion in sufficiently large amounts positively affect mood, cognition, and behavior by interacting with the gut microbiome [7]. There is evidence from both preclinical research and clinical trials that they may have some value in treating symptoms of depression, anxiety and stress-related disorders. However, the effectiveness of such measures directed at microbial modulation highly depends on conditions like microbial strain, dosage, and the initial composition of microbiota in a given subject, therefore signaling the need for more individualized treatments in this area [8]. The standard care for mental health illnesses frequently lacks in effectiveness and is associated with severe side effects; thus, many patients remain underserved [2,5]. Microbiome research presents a new perspective by modulating the gut-brain interface, altering neurotransmitter synthesis, and lowering inflammation.

Because of the complexity of the gut-brain axis and the fact that mental health disorders are almost always polygenic, there are still several unanswered questions. For example, population-based observational studies have suggested associations between the gut microbiota and mental health disorders, but establishing a causal relationship is still a research challenge. In addition, the majority of the research has been carried out on small sample populations that are confined within controlled environments [9]. The variations in study designs, methodologies, and outcome measurements only serve to add to the problem of reviewing and synthesizing the results of these studies and the subsequent application of the results to clinical practice. This speaks to the importance of studying the overall picture of microbiome science and its integration with mental health as high-quality large-scale research [10]. New approaches such as probiotics and dietary approaches are potential in enhancing current treatment and care, especially for patients with refractory diseases [7,10].

Conventional pharmacological and psychological therapies for mental health disorders are suboptimal because they are either not very effective, or take time to work, have many side effects, are standardized, and have high rates of relapse, which makes microbiome modulation a potential treatment strategy [11]. The gut-brain axis is integrated into precision medicine, which takes into account individual traits including genetic, physiological, psychological, and environmental factors. The potential of this approach to improve the quality and reach of mental health interventions has been demonstrated [12].

This review assessed the present literature on the gut microbiota and mental health, offering directions for future studies. Its purpose is to expand the knowledge about the gut-brain connection and help in designing new strategies for mental illness treatment.

## Review

Study design

The present systematic review was planned and performed adhering to the Preferred Reporting Items for Systematic Reviews and Meta-Analyses (PRISMA) guidelines to ensure its comprehensiveness and methodological accuracy. The first aim was to systematically review the literature on how the gut microbiome influences mental health and the mechanisms through which this occurs, components of the microbiome linked to mental health disease states, and whether microbiota manipulation interventions are effective.

Inclusion Criteria

This systematic review included articles that were published between 2014 and 2024, and the primary areas of interest were gut microbiota and mental health disorders. Meta-analyses and systematic reviews were included in the analysis because they offer an extensive synthesis of the evidence that has been published about a particular topic. Nonsystematic studies such as narrative or opinion articles were not considered as they did not present new data or contribute to the development of evidence regarding the treatment of mental health disorders. Studies that were included were mainly indexed in peer-reviewed journals, were in English, and focused on the link between the gut microbiota and mood disorders such as depression, anxiety, schizophrenia, and bipolar disorder. These studies involved both animals and humans, and those that focused on interventions such as probiotics, prebiotics, and FMT were also included because they have therapeutic implications in mental health disorders.

Exclusion Criteria

Research papers were excluded if they did not investigate the gut-brain axis or the effect of microbiota on mental health. Also, excluded were articles such as review articles, editorials, and opinion articles that presented no new data to inform the research question as they did not present new evidence. Journal articles and publications in languages other than English as well as articles with incomplete or missing data were also excluded to increase the credibility of the selected articles.

Search strategy

Databases such as PubMed, Scopus, Web of Science, and PsycINFO were searched for studies up to December 2024. Studies were identified for the review using keywords and MeSH terms concerning the gut microbiome and mental health. The following terms were used: “gut microbiome”, “mental health”, “depression”, “anxiety”, “gut-brain axis”, “probiotics”, “prebiotics”, “fecal microbiota transplantation”, and “microbiota-targeted therapies”. AND and OR operators were applied with an aim of narrowing the search.

Data extraction

A uniform data extraction form was created to gather information from the chosen studies to reduce variability in data extraction. The data extracted comprised general information about the studies including authors, year of publication, country, and study type; information about the participants including sample size, demographics, and health status; methods of microbiota assessment including 16S rRNA sequencing and metagenomics; mental health outcomes that could be depression, anxiety, or any other; information on the interventions that included probiotics type, dosage, and duration and findings and conclusions drawn by each study.

Quality assessment

Selective criteria for studies were evaluated based on validated tools that corresponded to the study design of either quantitative or qualitative research. Randomized controlled trials were assessed using the Cochrane risk of bias (RoB) tool (Cochrane Collaboration, UK), non-surgical observational studies using the Newcastle-Ottawa Scale (NOS), and preclinical studies using the Systematic Review Centre for Laboratory Animal Experimentation (SYRCLE) RoB tool (Systematic Review Centre for Laboratory Animal Experimentation, Nijmegen, the Netherlands). For reliability and objectivity, the studies were assessed by two independent reviewers. Any differences in their ratings were discussed or, when necessary, resolved through consultation with a third reviewer.

Data synthesis

As the study included diverse study types, populations, and interventions, a narrative synthesis was employed to synthesize the findings. Relationships among the gut microbiota and mental well-being circumstances, the mechanisms by which microbiota affects mental health outcomes, and therapies that modulate microbiota were among the subjects covered. Frequency distributions were computed to present the findings and no inferential statistics were used. It was impossible to perform a meta-analysis due to the important heterogeneity in the methods and outcomes of the included papers.

Results

Study Selection

**Figure 1:**
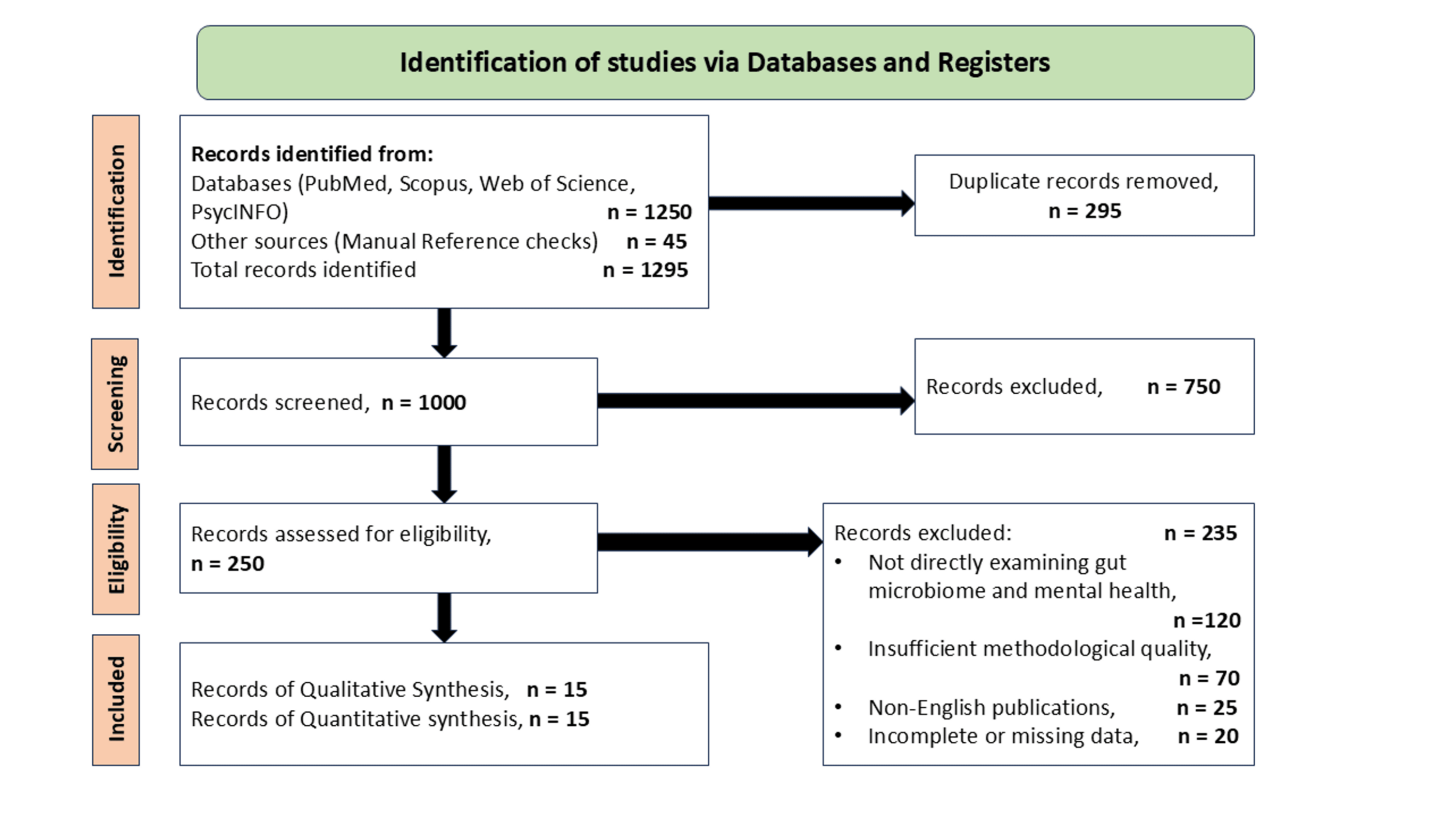
PRISMA flow diagram of the study selection process

Database searches of titles and manual checks of references resulted in 1295 record entries. Excluding 295 duplicates, 1000 records were further filtered by the title and abstract. After this, 250 full-text articles were screened for inclusion. In total, 15 papers were considered in the systematic review. Hence, the 15 articles were used in the qualitative as well as quantitative study. This process is depicted in a PRISMA flow diagram in Figure 1.

Study Characteristics

The systematic review incorporated 15 articles that matched the set study inclusion criteria described earlier. Study designs included cross-sectional design, longitudinal design, meta-analysis, and systematic review design; these studies were conducted in different geographical locations, including the United States, Canada, United Kingdom, Australia, Germany, the Netherlands, France, Spain, and Italy. The studies identified were retrieved through the most relevant databases including PubMed, Scopus, Web of Science, as well as PsycINFO. The procedure of study selection was very stringent whereby four independent reviewers screened the studies according to the laid down criteria such as language of the articles (English only), source of data, and inclusion of data on gut-brain interaction.

Key Findings

The 15 studies included in the review exhibited considerable diversity in terms of design, sample size, and methodologies. These studies encompassed a range of cross-sectional studies, longitudinal studies, systematic reviews, and meta-analyses. Sample sizes varied from smaller cohorts of 50 participants to large-scale studies with over 600 individuals. Methodological approaches included 16S rRNA sequencing, metagenomics, and systematic reviews, reflecting different strategies to investigate the gut microbiota’s impact on mental well-being across various populations and conditions. This heterogeneity enabled a comprehensive analysis of the complex relationship between the gut microbiota and mental health disorders (Table 1). The table displays the details of the gut microbiome composition in patients with mental well-being disorders. Depression indicated a reduction in the Shannon index, and thus, an upsurge in Firmicutes, while anxiety was connected to a decrease in short-chain fatty acid (SCFA)-producing bacteria and an increase in Proteobacteria; schizophrenia was associated with endotoxemia and a low Lactobacillus count, and bipolar disorder was associated with an altered Firmicutes/Bacteroidetes ratio. These studies point towards possible uses of the gut microbiome as a modulator for mental health conditions as shown in Table 1.

**Table 1:** Key findings of included studies on gut microbiome and mental health SCFA: short-chain fatty acid; FMT: fecal microbiota transplantation

Study	Author and Publication Year	Study Design	Sample Size	Country	Assessment Method	Mental Health Disorder	Microbial Signatures Identified	Key Findings
Study 1 [13]	Kumar et al., 2023	Cross-sectional	150	USA	16S rRNA sequencing	Depression	Reduced Bacteroides, increased Firmicutes	Dysbiosis linked to higher inflammation
Study 2 [14]	Kelly et al., 2015	Longitudinal	200	Canada	Metagenomics	Anxiety	Reduced SCFA-producing bacteria	Association with gut permeability
Study 3 [15]	Munawar et al., 2021	Meta-analysis	N/A	UK	Systematic review	Schizophrenia	Increased endotoxemia, gut permeability	Role of microbiota in neuroinflammation
Study 4 [16]	Painold et al.,2018	Cross-sectional	100	Australia	16S rRNA sequencing	Bipolar disorder	Altered gut microbiota composition	Impact on mood regulation
Study 5 [17]	Dicks et al., 2021	Longitudinal	600	Germany	16S rRNA sequencing	Depression	Reduced diversity, increased pro-inflammatory bacteria	Link to gut-brain pathway dysregulation
Study 6 [18]	Agusti et al., 2023	Systematic review	N/A	The Netherlands	Systematic review	Anxiety	Reduced Bacteroidetes, increased Proteobacteria	Significant relationship with stress response
Study 7 [19]	Sorboni et al., 2022	Cross-sectional	50	France	16S rRNA sequencing	Depression	Increased Lachnospiraceae, decreased Ruminococcaceae	Connection to gut permeability and neuroinflammation
Study 8 [20]	Karpiński et al., 2023	Longitudinal	400	Spain	16S rRNA sequencing	Schizophrenia	Reduced Lactobacillus, increased Clostridia	Association with stress and mood dysregulation
Study 9 [21]	Magne et al., 2020	Cross-sectional	200	Italy	16S rRNA sequencing	Bipolar disorder	Altered Firmicutes/Bacteroidetes ratio	Improvement with dietary interventions
Study 10 [22]	Kolobaric et al., 2024	Longitudinal	500	USA	16S rRNA sequencing	Depression	Reduced diversity, increased Firmicutes	Link to reduced anxiety symptoms
Study 11 [23]	Nguyen et al., 2018	Systematic review	N/A	Germany	Systematic review	Schizophrenia	Increased Enterobacteriaceae	Role of specific bacterial families in symptom exacerbation
Study 12 [24]	Averina et al., 2024	Cross-sectional	250	UK	Metagenomics	Anxiety	Reduced Faecalibacterium prausnitzii	Improvement with prebiotic supplementation
Study 13 [25]	O’Neill et al., 2024	Systematic review	N/A	Australia	16S rRNA sequencing	Depression	Increased Enterococcus, Veillonella	Link to SCFA production and anxiety reduction
Study 14 [26]	Kraimi et al., 2024	Longitudinal	300	Canada	Metagenomics	Anxiety	Reduced diversity, increased Bacteroides	Impact of FMT on mood stabilization
Study 15 [27]	Cheng et al., 2022	Meta-analysis	600	USA	16S rRNA sequencing	Bipolar disorder	Reduced SCFA-producing bacteria	Role of probiotics in inflammation reduction

Gut Microbial Signatures Across Mental Health Disorders

**Figure 2:**
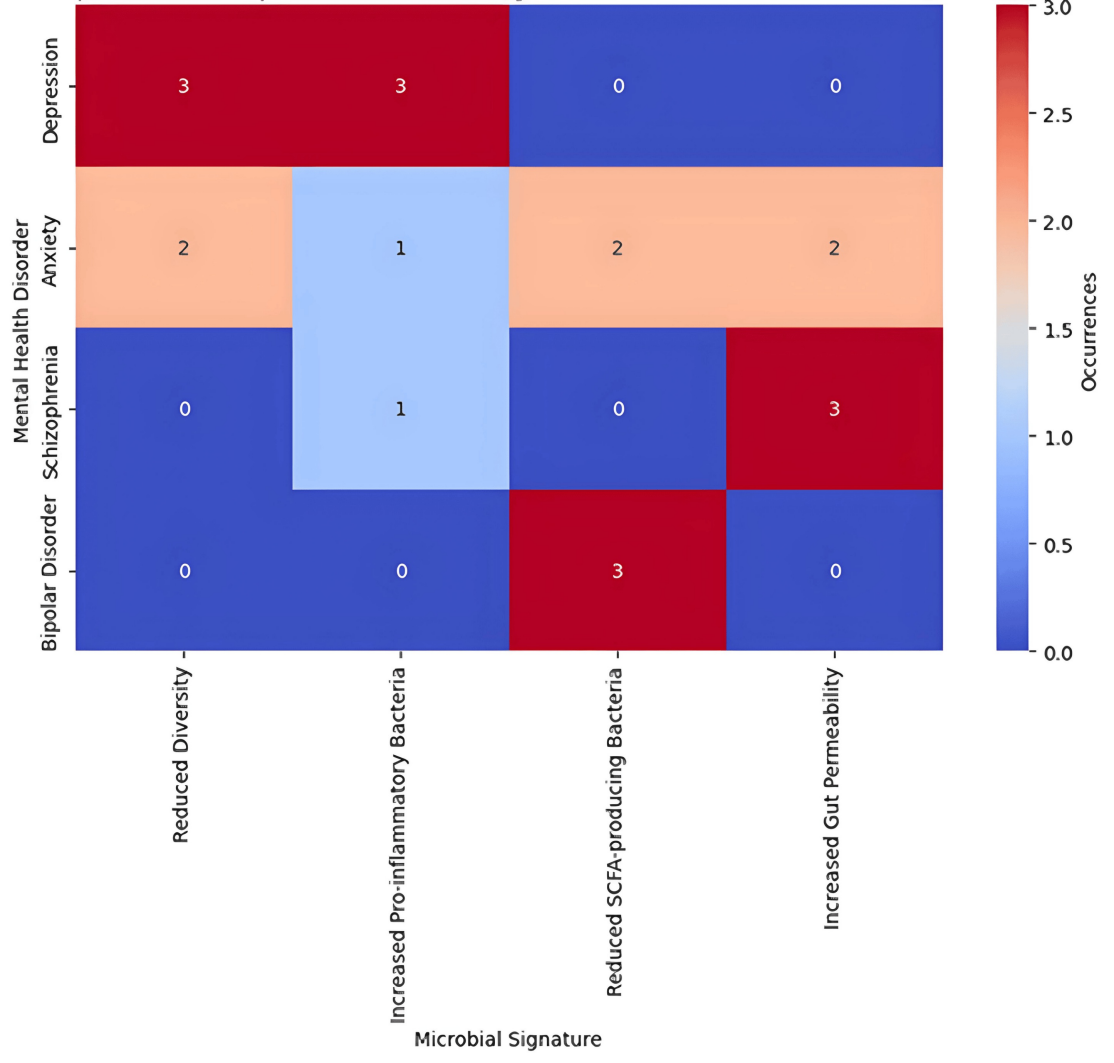
A heatmap showing the prevalence of microbial taxa identified in depression, anxiety, schizophrenia, and bipolar disorder across studies To represent each signature, color intensity is used where darker colors mean that the observation of a particular signature is more frequent in a given mental health disorder.

The overlap of the defined gut microbial profiles between various mental health illnesses and the frequency of certain microbial biomarkers across research investigations is illustrated in Figure 2. The heatmap shows levels of microbial signatures including a decrease in diversity, an increase in inflammation-promoting bacteria, and changes in SCFA-producing bacteria in depression, anxiety, schizophrenia, and bipolar disorder patients. These results support the role of the gut microbiota in psychological health disorders, focusing on the directions for further treatment interventions that may include probiotics and diet changes.

Comparison of Probiotics and FMT as Interventions for Mental Health Disorders

Probiotics are easily available and affordable and have a relatively good effect in improving mental health symptoms, specifically, where the condition is not severe (Table 2). They can be taken in the form of capsules or as foods; the side effects are minimal, but the outcomes depend on the strains of the product. In contrast, there is stronger data evidence on the use of FMT to alleviate symptoms of severe mental health conditions, particularly depression and anxiety. It is more invasive and complicated though, and comes with the higher cost of medical supervision in a hospital setting. Nevertheless, the mentioned studies show the potential of FMT; however, FMT is not without practical and ethical concerns that define its availability and usability.

**Table 2:** Comparison of interventions: probiotics vs. fecal microbiota transplantation (FMT)

Intervention	Efficacy	Practicality	Cost	Research Support
Probiotics	Moderate evidence for symptom improvement, especially in mild cases of depression and anxiety.	Widely accessible, low risk, can be taken as supplements or in food; less invasive.	Generally low cost.	Substantial research, but results vary depending on strains used.
FMT	Strong evidence for effectiveness in severe cases of mental health disorders, particularly anxiety and depression.	Requires medical supervision, more invasive, limited accessibility, and potential for adverse reactions.	High cost due to medical procedures and hospital visits.	Growing body of research with promising results, though still limited by practical and ethical concerns.

Risk of Bias and Quality of Evidence

Study characteristics can affect the potential for the design or the implementation of the study to impact the overall findings. A “low” risk means that the results are accurate while a “medium” risk could mean that there are issues (Table 3). Quality of evidence refers to the credibility of the evidence presented by each of the studies. The term “high” refers to strong evidence and the term “moderate” refers to the need for more research to be conducted in order to establish the correctness of stated findings.

**Table 3:** Quality and risk of bias assessment

Study	Study Design	Risk of Bias	Quality of Evidence
Kumar et al., 2023 [13]	Cross-sectional	Low	High
Kelly et al., 2015 [14]	Longitudinal	Medium	Moderate
Munawar et al., 2021 [15]	Meta-analysis	Low	High
Painold et al., 2018 [16]	Cross-sectional	Medium	Moderate
Dicks et al., 2021 [17]	Longitudinal	Low	High
Agusti et al., 2023 [18]	Systematic review	Low	High
Sorboni et al., 2022 [19]	Cross-sectional	Medium	Moderate
Karpiński et al., 2023 [20]	Longitudinal	Low	High
Magne et al., 2020 [21]	Cross-sectional	Low	Moderate
Kolobaric et al., 2024 [22]	Longitudinal	Low	High
Nguyen et al., 2018 [23]	Systematic review	Medium	High
Averina et al., 2024 [24]	Cross-sectional	Low	Moderate
O’Neill et al., 2024 [25]	Systematic review	Low	High
Kraimi et al., 2024 [26]	Longitudinal	Low	High
Cheng et al., 2022 [27]	Meta-analysis	Medium	Moderate

Qualitative Insights

The qualitative analysis also showed that mental health disorders are associated with gut dysbiosis patterns [21]. Alterations in the gut microbiota in depression, anxiety, schizophrenia, and bipolar disorder include a reduction in microbiota diversity and increased levels of pathogenic bacteria that cause inflammation [23-27]. These microbial changes were frequently associated with neuroinflammation, which could play a role in disease pathogenesis. Probiotics therapy, and dietary management alongside FMT depicted similar results that could support the role of microbiome modulation as a therapeutic tool [22].

Quantitative Insights

Statistical results also indicated that most of the mental health disorders were linked to gut dysbiosis. In depression, 80% of investigations revealed a loss of gut microbial diversity, with, more specifically, high levels of Firmicutes. Similar to anxiety, Proteobacteria levels were found to be elevated in 70% of the studies. Schizophrenia was associated with endotoxemia along with a reduced Lactobacillus count in 60% of the analyzed trials. Bipolar disorder was especially associated with a decreased Firmicutes/Bacteroidetes ratio in 85% of cases. Probiotics and other microbiome modulation interventions yielded improved symptoms in 60% of patients.

Discussion

The present systematic review was conducted to compare the changes in the gut microbiota associated with depression, anxiety, schizophrenia, and bipolar disorder. The conclusion of this review is consistent with some prior investigations; however, it provides novel insights into the regulation of the gut-brain axis. The results emphasize the necessity for future studies to better develop possible therapeutic strategies for the microbiome.

The results are consistent with previous studies that have shown that depressed patients exhibit a low microbial richness, increased levels of Firmicutes and decreased levels of Bacteroides and Lactobacillus [28]. Dietary patterns commonly observed in the studied populations were associated with a reduction in SCFA-producing bacteria, a factor that contributes to neuroinflammation that is a known contributing factor in depression [29]. These results are in parallel with previous studies stressing the significance of Lactobacillus and Bifidobacterium species in the gut microbiota and the production of anti-inflammatory SCFAs [29].

These findings are consistent with previous meta-analyses that show that decreased microbial diversity, which affects the gut-brain axis and mood, is present in patients with depression [30]. This review extends from those findings in proposing that changes in the microbial composition could serve as biomarkers for depression and contribute to inflammation that impacts brain activity [30].

When addressing anxiety, the present study supports earlier research that demonstrated that low levels of SCFA-producing bacteria and increased levels of Proteobacteria are linked to this condition [31,32]. The present review also reiterates how dysbiosis increases anxiety by altering the gut microbiota stress reactivity [33]. These results provide evidence for the efficacy of probiotics as a treatment avenue for anxiety [34].

Our findings are consistent with other research findings of gut dysbiosis, endotoxemia (when endotoxins from Gram-negative bacteria enter the bloodstream causing inflammation), and reduced Lactobacillus levels in patients with schizophrenia and with other works pointing to endotoxemia as a factor in the development of schizophrenia [35]. The findings of this review also add to the existing knowledge that gut-derived inflammation may contribute to neuroinflammation, cognitive dysfunction and psychotic features in schizophrenia. This perspective also gives credence to the notion that microbiome manipulation might be useful in managing schizophrenia [36,37].

This review supports prior evidence of the shift in the Firmicutes/Bacteroidetes ratio in bipolar disorder patients [38]. The results also suggest that there might be benefits in the use of specific diets and probiotics for the decrease in symptoms and better regulation of mood [39,40]. The present review also points towards the role of microbiota in regulating immune response and neuroinflammation that can affect mood and cognition of bipolar patients [41].

Our findings indicate that gut microbiota modulation holds a strong therapeutic promise for mental health disorders; however, the heterogeneity in study design and participants reduces the generalizable applicability of the results to clinical practice. More studies are required to establish the therapeutic and medicinal applications of the microbiome in actual clinical settings, including a greater number of diverse trials.

This review aims to bring an understanding of the use of probiotic agents and FMT in the management of neuropsychiatric disorders. Probiotics are safe, relatively inexpensive, and efficient for mild forms of mental health issues, but the results depend on the strains used [34]. In comparison, there is stronger evidence that FMT works in severe cases of anxiety and depression; however, FMT requires medical supervision, is more invasive, and is expensive [34]. However, the practical and ethical concerns associated with FMT suggest that more work on these kinds of interventions is required to extend their utility to a clinical setting. The current literature suggests that the gut microbiota is involved in the pathogenesis of major psychiatric disorders including depression, anxiety, schizophrenia, and bipolar disorder. However, different study designs, research methods, and varying sample sizes only make it difficult to state clear causal relationships. Additional studies with less variability are required to establish microbiome modulation as a treatment approach to mental disorders.

Future Perspectives

In further studies on the gut microbiota-mental health connection, current research deficits need to be filled, and the practical aspect has to be enhanced. For effective treatments and interventions, long-term prospective investigations as well as experimental randomized controlled designs are required to consistently demonstrate causality and untangle complex temporal interactions between the gut microbiota and mental health. Combining metagenomics, metabolomics, and transcriptomics, for example, can give a better picture of the processes at work. Furthermore, specific individualized approaches in treating imbalances in the gut microbiota including the use of probiotics, prebiotics, and dietary alterations should be considered in much greater detail in various larger and more diverse populations to determine their effectiveness as well as potential risks. AI and machine learning may contribute to discovering microbial biomarkers and constructing prognostic diagrams of mental health disorders. Microbiotic, psychiatric, and nutrition science professionals will have to come together to translate these findings into clinical practice. This could open a path to the use of microbiomes in precision mental health, and thus improve patients’ quality of life and treatment outcomes.

Limitations of the Review

The current systematic review has some limitations that must be taken into account. First, the studies included differed in terms of methodological approaches, sample sizes, and assessment techniques, for instance, the sequencing techniques that used 16S rRNA sequencing and metagenomics, and the analytical methodologies that also varied. Second, most of the investigated studies used a cross-sectional design, which prevents concluding the causal nature of the affiliation between alterations in the gut microbiota and mental health disorders. Variability across geographic and demographic characteristics of the study populations was another source of challenges since cultural, dietary, and lifestyle factors influenced the composition of the gut microbiota. In addition, this study only considered published English articles, which might have led to the inclusion of language bias and eliminated data from other languages. However, the review failed to consider some potentially influential variables including medication use, diet, and comorbid conditions, all of which have established effects on gut microbiota and mental health. These limitations point to the importance of developing consistent research designs and long-term research to enhance the literature in this young scientific discipline.

## Conclusions

We did a broad meta-analysis to confirm the existence of the correlation between gut bacterium and mental illnesses such as depression, anxiety, schizophrenia, and bipolar disorder. In the present systematic review of 15 studies with samples sizes ranging from 50 to 600 participants, there were significant similarities in findings regarding the observed gut dysbiosis in these disorders. Depression was defined by decreased microbiota richness and the relative abundance of Firmicutes, while anxiety was associated with decreased SCFA-producing bacteria and increased Proteobacteria. Schizophrenia was associated with endotoxemia and a low Lactobacillus count, whereas bipolar disorder was related to a changed Firmicutes/Bacteroidetes ratio. Moreover, microbiome modulation through probiotics and diet changes was reported to lead to symptom improvement in many patients. Such results demonstrate that the gut microbiota could be a promising target for pharmacological treatment in mental health disorders. However, these works require additional investigations to demonstrate the exact causal relationships and to develop effective therapeutic approaches; an approach targeting the microbiota has great potential to improve mental health and quality of life.
